# Editorial: Perturbation-based balance training

**DOI:** 10.3389/fspor.2023.1306133

**Published:** 2023-10-19

**Authors:** Christopher McCrum, Yoshiro Okubo

**Affiliations:** ^1^ Department of Nutrition and Movement Sciences, NUTRIM School of Nutrition and Translational Research in Metabolism, Maastricht University, Maastricht, Netherlands; ^2^ Falls, Balance and Injury Research Centre, Neuroscience Research Australia, Sydney, NSW, Australia; ^3^ Faculty of Medicine and Health, University of New South Wales, Sydney, NSW, Australia

**Keywords:** balance, falls, gait, perturbations, slips, stability, trips

**Editorial on the Research Topic** Perturbation-based balance training

## Introduction

Perturbation-based balance training (PBT; or reactive balance training or perturbation training) is balance training that uses repeated, externally applied mechanical perturbations to trigger rapid reactions to regain postural stability in a safe and controlled environment (McCrum et al.). The goal of PBT is to specifically target and improve the ability to maintain and recover balance in situations that often lead to falls. There is evidence suggesting that perturbation-based balance training can reduce falls in everyday life by up to 40%–50% (1–3). This is particularly promising given the relatively short time needed to achieve these benefits, in comparison to traditional exercise programs. However, there were and are important knowledge gaps for this approach to fall prevention, especially regarding its efficacy, mechanisms, optimal dose, type and presentation of perturbation, transfer or generalisability to daily life tasks, application/feasibility in various clinical populations and retention of the improvements over time. Therefore, this Research Topic aimed to collect contributions on the latest developments related to perturbation-based balance training. Contributions could be broadly classified into three categories: (i) Balance measures using perturbations, (ii) effects and mechanisms and (iii) implementation of perturbation-based balance training.

## Balance measures using perturbations

Five articles in this Research Topic contributed insight into assessing balance during perturbations. Two articles presented tests using instrumented treadmills: Lesch et al. proposed a perturbed postural balance test using an instrumented treadmill which has become increasingly common in biomechanics laboratories and clinical settings instead of purpose-built movable force plates; and Adams et al. proposed the Stepping Threshold Test using an instrumented treadmill and observation of stepping behaviours via video recording. Two studies examined specific outcomes derived from perturbation testing: Rieger et al. proposed a simple way to track balance recovery performance during gait perturbation training using the center of pressure data from an instrumented treadmill; and Gerards et al. demonstrated that adaptability to gait perturbation via treadmill belt accelerations was related to history of falls in older adults. Finally, in a comprehensive overview, Grabiner and Kaufman reviewed the literature and stated the need for developing and establishing biomechanical risk biomarkers for preventable falls such as those induced by trips. They proposed trunk kinematics as a biomarker for trip-specific falls.

## Effects and mechanisms of PBT

Seven articles in this Research Topic addressed various mechanisms and effects of PBT. Two specifically addressed transfer between different tasks. A randomised cross-over trial by Song et al. directly compared acute motor adaptations to commonly used treadmill belt accelerations trips against obstacle trips on a walkway. They reported that older adults could learn to improve dynamic stability by repeated exposure to both perturbation modalities, but the adaptations to treadmill belt accelerations did not transfer to an actual trip. In slight contrast, Bhatt et al. compared groups of older adults who completed slip or trip training following novel perturbations of the untrained type. The training resulted in proactive adjustments that could worsen the reactive response to the opposite perturbation (interference) but older adults could generalise their improved reactive control to maintain dynamic stability (margin of stability), to preserve limb support control, and to reduce fall risk.

Four articles addressed aspects relating to muscular contributions and kinetics of balance recovery. Yoo et al. elucidated the kinetic and muscular mechanisms of balance recovery following a split-belt treadmill perturbation. Older people showed greater joint moments and muscle responses of the compensatory limb during the recovery period than in younger people. In contrast, older people showed greater co-contraction of biceps femoris/rectus femoris muscles during recovery, likely compensating for their muscle weakness. Debelle et al. studied kinematic and kinetic mechanisms of improved balance recovery to repeated backward slips simulated by treadmill belt accelerations in older and younger adults. Regardless of age, dynamic stability improved with repeated exposure, which was related to change in step length and ground reaction force angle. Staring et al. investigated kinematic and muscular mechanisms of improved stepping responses to backward and forward platform translations which were repeatedly applied to chronic stroke survivors. Although muscle onset became faster in gastrocnemius and likely in tibialis anterior, these were not related to increase in step length, duration and velocity which were related to a more upright position. Van Wouwe et al. compared the effects of a traditional 12-week resistance exercise program and a 3-week PBT program using support-surface perturbations of stance in older adults. The study found intervention-specific effects (improved strength due to resistance exercise and improved reactive balance during stance perturbations due to the PBT) and reported that muscle strength was not a limiting factor for reactive balance. However, neither intervention translated to improved performance of perturbation recovery during walking.

One final study was potentially a first for the field, in which Martelli et al. trained older adults using waist-pull perturbations on a treadmill. This study showed that a single session of perturbation-based balance training produces acute aftereffects in terms of increased cognitive performance and gait stability in healthy older adults.

## Implementation

The final three articles of the Research Topic considered some implementation-related aspects of PBT. As a major barrier of PBT is the limited accessibility to perturbation equipment and a safety harness, Lee et al. proposed a novel manual technique for trip recovery training. Another gap in the current literature is how psychological factors play a role in the effectiveness of PBT and the article by Soh provides a reasoned overview of ways to measure and interpret falls efficacy, balance confidence, and balance recovery confidence in this context. Finally, in our review article McCrum et al. we provide a definition of PBT as “balance training that uses repeated, externally applied mechanical perturbations to trigger rapid reactions to regain postural stability in a safe and controlled environment.” and discuss the current state of research on PBT from the perspectives of the basic principles, mechanisms and implementation in practice.

## Conclusions

PBT is a promising approach to fall prevention. With each year, more studies provide insight into both the underlying mechanisms of this training and how to better implement it in practice. However, as we noted in McCrum et al. several fundamental and applied aspects of PBT still need to be investigated and understood in order for it to be widely and successfully applied in practice.
